# Anti-Inflammatory Effects of *Lactiplantibacillus plantarum* Strain FS4722 Through MAPK and NF-κB Signaling Pathways and Its Lyophilization Optimization

**DOI:** 10.3390/foods15061096

**Published:** 2026-03-20

**Authors:** Bista Sunita, Yuxing Liu, Hanwei Zheng, Yue Su, Mingyue Liu, Linfeng Xu, Ikram Alouk, Zhiqing Liu, Wenyong Lou

**Affiliations:** Lab of Applied Biocatalysis, School of Food Science and Engineering, South China University of Technology, No. 381 Wushan Road, Guangzhou 510640, China; sunitabista2244@gmail.com (B.S.); 18198451030@163.com (Y.L.); 13527890061@163.com (H.Z.); 18138017700@163.com (Y.S.); lmy1306@163.com (M.L.); 18316418233@163.com (L.X.); aloukikram@yahoo.fr (I.A.)

**Keywords:** probiotics, *Lactiplantibacillus plantarum* FS4722, anti-inflammatory activity, MAPK and NF-κB, freeze-drying protective

## Abstract

Probiotics hold considerable promise for treating and preventing inflammatory disease; however, their application is often limited by unclear anti-inflammatory mechanisms and reduced viability following lyophilization. In this study, I thoroughly evaluated the anti-inflammatory potential of *Lactiplantibacillus plantarum* FS4722 (*L. plantarum* FS4722) and substantially enhanced strain viability through optimization of the lyoprotectant formulation. Functional assays demonstrated that the fermented supernatant, heat-inactivated bacterial suspension, and cell lysate derived from *L. plantarum* FS4722 effectively suppressed transcription and expression of inflammatory cytokines in LPS-stimulated RAW 264.7 macrophages. The fermented supernatant exhibited the strongest inhibitory effects, surpassing the reference probiotic *Lacticaseibacillus rhamnosus* GG (LGG). Mechanistic investigations revealed that anti-inflammatory activity is primarily mediated via inhibition of the MAPK and NF-κB signaling pathways. Furthermore, using component screening combined with response surface methodology, the lyoprotectant formulation (10.00% trehalose, 1.00% sodium carboxymethyl cellulose, and 5.00% skim milk) was optimized, resulting in a lyophilization survival rate of 82.32% while maintaining cellular integrity; in this accelerated stability assessment, the strain retained 78.89% of its activity after 28 days of storage at 4 °C. Collectively, this study provides a robust and efficient approach for probiotic formulation while systematically elucidating the underlying anti-inflammatory mechanisms, thereby offering practical guidance for the development and clinical application of high-performance probiotic products.

## 1. Introduction

In response to infection, tissue damage, or stress, inflammation serves as an essential defense mechanism that involves the coordinated regulation of multiple immune cell populations and complex signaling pathways [1]. Key signaling pathways, such as NF-κB and MAPK, are activated when pathogen-associated or damage-associated molecular patterns are recognized by pattern recognition receptors (e.g., TLRs, NLRs), resulting in the strong release of pro-inflammatory cytokines (e.g., TNF-α, IL-1β, IL-6) [2]. Rheumatoid arthritis, inflammatory bowel disease, atherosclerosis, and cancer are among the disorders whose pathophysiology is intimately linked to dysregulation or excessive activation of these pathways [3,4]. Non-steroidal anti-inflammatory medications, corticosteroids, biologics (such as anti-TNF-α antibodies), and small-molecule inhibitors that target certain pathways are the mainstays of current clinical anti-inflammatory therapies [5]. Long-term use of these medications frequently results in serious side effects such as immunosuppression, metabolic disorders, and organ toxicity, despite their effectiveness in reducing symptoms and slowing the progression of disease [6]. Furthermore, some patients show an insufficient response or treatment resistance [7]. Thus, creating safer, more personalized and targeted anti-inflammatory strategies remains a significant challenge.

Probiotics have attracted considerable attention lately due to their diverse immunomodulatory properties. Probiotics, such as *L. plantarum*, *Bifidobacterium* spp., and *Limosilactobacillus fermentum*, have been shown to preserve intestinal barrier integrity, competitively inhibit pathogen colonization, and modulate immune cell functions [8]. This, in turn, regulates inflammatory signaling pathways such as NF-κB and MAPK, promoting immune homeostasis and exerting anti-inflammatory effects [9]. Because of its notable effectiveness in regulating gut immunity and anti-inflammatory responses, *L. plantarum* has become a study focus [10]. In models of inflammatory bowel disease and metabolic inflammation, prior research has shown that *L. plantarum* significantly reduces the expression of pro-inflammatory cytokines like TNF-α, IL-6, and IL-1β while promoting anti-inflammatory cytokines like IL-10. They also increase macrophage phagocytic activity, suppress the activation of inflammatory signaling pathways, and maintain epithelial barrier homeostasis [11,12].

However, turning these anti-inflammatory benefits into drugs that may be used in therapy requires good ways to keep them safe. Lyophilization is the greatest approach to keep probiotics alive for a long time. It makes it easy to store, share, and preserve their metabolic activity [13]. This method, on the other hand, puts germs through a lot of stress, like cold temperatures, dehydration, and damage from machines. Without proper protection, this can hurt bacterial membranes, disrupt proteins, and make bacteria less able to live by 40–70% [14]. Because probiotics lose their ability to work after lyophilization, they are still challenging to employ in business. This is a huge concern because it implies they work in the lab but not in the real world [13,14].

To solve this problem, optimization alternatives presently include improving protectant formulations, coming up with new delivery systems, making pretreatment procedures easier, and applying synthetic biology technologies [14,15]. People think that enhancing the formulas of protectants is the easiest and best way to go. Cryoprotectants reduce cellular damage during lyophilization and significantly improve bacterial recovery and functional preservation through mechanisms such as osmotic control, free radical scavenging, and membrane protection [16]. Choosing the right lyoprotectants is particularly crucial for keeping immunomodulatory activity since the stability of proteins and the integrity of membranes directly affect how the host recognizes pathogens and generates signaling molecules.

The goal of this work is to fill in this gap by integrating molecular studies of anti-inflammatory effect with practical formulation improvement for *L. plantarum* FS4722. To clarify the mechanisms involved and confirm anti-inflammatory activity, I investigated the impacts of fermented supernatant, inactivated bacterial cells, and cell lysates on the synthesis of inflammatory mediators and evaluated their influence on MAPK and MyD88/NF-κB signaling pathways using an LPS-induced RAW 264.7 macrophage model. Second, I carefully examined and modified the lyoprotectant formulations so that they would be more stable when stored and better able to survive freeze-drying. I got both biological proof and technical help for future industrial usage from this. This two-part plan makes sure that the anti-inflammatory effect seen in vitro may be kept and used in a clinical situation.

## 2. Materials and Methods

### 2.1. Bacterial Strains

*L. plantarum* FS4722, initially isolated from paocai, a traditional Chinese fermented meal, was sourced from a laboratory culture collection where it had been maintained. Li et al. [17] earlier reported their first screening and the capacity to decrease uric acid. The current work aims to further evaluate its probiotic qualities. The commercial probiotic reference strain LGG was sourced from the Beijing Beina Culture Collection Center, (Beijing, China) [18]. We got all of the lactic acid bacteria strains from frozen stocks and grew them in MRS broth (BD Biosciences, San Jose, CA, USA) at 37 °C for 18 h (Waltham, MA, USA) without oxygen. We then subcultured each strain twice in a row to get metabolically active cultures for future investigations.

### 2.2. Sample Preparation

*L. plantarum* FS4722 Fermented Supernatant (FS4722-Fs): The first culture was altered to a cell density of 9 log CFU/mL by adding sterile distilled water that had been prepared with a Milli-Q water purification system (Millipore, St. Louis, MO, USA). We spun the suspension at 4 °C and 10,000× *g* (Implen, Munich, Germany) for 15 min. We took the supernatant and filtered it through a Millipore (St. Louis, MO, USA) 0.22 μm polyvinylidene fluoride (PVDF) membrane. The filtrate that came out was the fermented supernatant. We added the supernatant to high-glucose Dulbecco’s Modified Eagle Medium (Gibco, Grand Island, NY, USA) to make a final concentration of 20% (*v*/*v*) for the cell intervention experiments.

*L. plantarum* FS4722 Heat-Killed Bacterial Suspension (FS4722-Dbs) and LGG Heat-Killed Bacterial Suspension: In an 80 °C water bath (Shanghai Jinghong Laboratory Instrument Co., Ltd., Shanghai, China), the first cultures were killed for half an hour. After that, they were spun at 10,000× *g* for 15 min. We threw away the supernatant and rinsed the bacterial pellet with phosphate-buffered saline (Gibco, Grand Island, NY, USA). We did this washing and spinning procedure three times. The pellet was resuspended in full high-glucose DMEM to a concentration of 9 log CFU/mL based on previous colony count results. An aliquot of the suspension was put on MRS agar and kept at 37 °C for 48 h to make sure that all the bacterial cells were dead.

*L. plantarum* FS4722 Bacterial Lysate (FS4722-Bl): The first culture was spun for 15 min at 4 °C and 10,000× *g*. The supernatant was thrown away, and the bacterial pellet was washed three times with PBS by spinning it in a centrifuge. The pellet was then placed back into DMEM with a high glucose level of 9 log CFU/mL. A JY92-IIDN ultrasonic cell disruptor (Ningbo Scientz Biotechnology Co., Ltd., Ningbo, China) was used to break up the suspension at 40 kHz for 15 min while it was on ice. Optical microscopy (Olympus, Tokyo, Japan) verified that the bacterial cells were completely broken down. We spun the lysate at 4 °C and 10,000× *g* for 15 min. The supernatant that came out was the bacterial lysate.

### 2.3. Cell Line and Culture Conditions

RAW 264.7 cells, a murine macrophage cell line, were obtained from Wuhan Pricella Biotechnology Co., Ltd. (Wuhan, China). The cells were cultured in Dulbecco’s Modified Eagle Medium (Gibco, Grand Island, NY, USA) supplemented with 10% (*v*/*v*) fetal bovine serum (FBS; Gibco) and 1% (*v*/*v*) penicillin–streptomycin solution (Gibco). All cells were maintained in an incubator at 37 °C with 5% CO_2_. When cell confluence reached 70–80%, subculturing was performed using 0.25% trypsin–EDTA solution (Gibco).

### 2.4. Assessment of Cell Viability of FS4722-Fs, FS4722-Dbs, and FS4722-Bl

Cell viability was evaluated using the 3-(4,5-dimethylthiazol-2-yl)-2,5-diphenyltetrazolium bromide (MTT; Sigma-Aldrich, St. Louis, MO, USA) assay to determine the effects of FS4722-Fs, FS4722-Dbs, and FS4722-Bl on RAW 264.7 cells. The assay was conducted following a previously established protocol [19], with minor modifications. The sample size (n = 3) was established based on standard methodology for in vitro mechanistic studies, ensuring sufficient statistical power (≥80%) to detect effect sizes of ≥20% at α = 0.05, consistent with analogous research in the field. In brief, RAW 264.7 cells (2 × 10^5^ cells/well) were seeded into 96-well plates (Corning, Corning, NY, USA) and incubated for 2 h, followed by treatment with the indicated samples and further incubation for 24 h. Subsequently, the culture supernatant was removed, and MTT solution was added to each well to a final concentration of 0.5 mg/mL. The plates were then incubated at 37 °C for 1 h. After careful removal of the MTT solution, the formazan crystals were dissolved in dimethyl sulfoxide (St. Louis, MO, USA). Absorbance at 570 nm was measured using a microplate spectrophotometer (Thermo Fisher Scientific, Waltham, MA, USA) after shaking the plates in the dark at room temperature for 15 min.

### 2.5. Quantification of NO Production

Nitric oxide (NO) production in lipopolysaccharide (LPS)-stimulated RAW 264.7 cells was determined using the Griess reagent method as previously described [20] to evaluate the effects of FS4722-Fs, FS4722-Dbs, and FS4722-Bl. Briefly, RAW 264.7 cells (2 × 10^5^ cells/well) were seeded into 96-well plates (Corning) and incubated for 2 h, followed by treatment with the indicated samples for an additional 2 h. Two control groups were included: a negative control without LPS and a positive control treated with LPS (1 μg/mL; Sigma-Aldrich). The cells were then further incubated for 24 h. Subsequently, the culture supernatants were collected and mixed with an equal volume of Griess reagent (Beyotime, Shanghai, China). After shaking, the mixtures were incubated in the dark for 15 min, and the absorbance at 540 nm was measured using a microplate spectrophotometer (BioTek, Shoreline, WA, USA).

### 2.6. Evaluation of PGE2 and Inflammatory Cytokines

The effects of FS4722-Fs, FS4722-Dbs, and FS4722-Bl on the production of prostaglandin E2 (PGE2) and the inflammatory cytokines IL-6, TNF-α, and IL-10 in LPS-stimulated RAW 264.7 cells were determined using enzyme-linked immunosorbent assay (ELISA). PGE2 levels were measured using a commercial ELISA kit (Nanjing Jiancheng Bioengineering Institute, Nanjing, China), while mouse IL-6, TNF-α, and IL-10 levels were quantified using corresponding ELISA kits from the same manufacturer. RAW 264.7 cells (5 × 10^5^ cells/well) were seeded into 12-well plates (Corning) and maintained at 37 °C in a humidified atmosphere containing 5% CO_2_ for 2 h. The cells were then treated with FS4722-Fs, FS4722-Dbs, or FS4722-Bl for 2 h, followed by stimulation with LPS for 24 h. A control group without LPS treatment was included. Subsequently, the culture supernatants were collected, appropriately diluted, and processed according to the manufacturer’s instructions. Absorbance was measured using a microplate spectrophotometer (BioTek), and the concentrations of PGE2, IL-6, TNF-α, and IL-10 were calculated based on standard curves.

### 2.7. Evaluation of Inflammatory Cytokine Transcriptome Expression in LPS-Activated RAW 264.7 Cells Treated with FS4722-Fs, FS4722-Dbs, and FS4722-Bl

The transcript expression of inflammatory cytokines in LPS-stimulated RAW 264.7 cells was analyzed by real-time polymerase chain reaction (RT–PCR) based on a previously reported method [21] with minor modifications. RAW 264.7 cells (1 × 10^6^ cells/well) were seeded into six-well plates (Corning) and maintained at 37 °C in a humidified atmosphere containing 5% CO_2_ for 24 h. The cells were then treated with FS4722-Fs, FS4722-Dbs, or FS4722-Bl for 2 h, followed by stimulation with LPS (1 μg/mL) for 24 h.

Total RNA was extracted using TRIzol reagent (Thermo Fisher Scientific), and RNA purity and concentration were determined using a NanoPhotometer^®^ N60 microvolume spectrophotometer (Implen, Munich, Germany). Subsequently, 1 μg of total RNA was reverse-transcribed into cDNA using the SensiFAST™ cDNA Synthesis Kit (Nanjing Jiancheng Bioengineering Institute). Quantitative real-time PCR (qRT-PCR) analysis was performed using the SensiFAST™ SYBR No-ROX Kit (Nanjing Jiancheng Bioengineering Institute) with SYBR Green dye on a QuantStudio™ 1 Real-Time PCR System (Thermo Fisher Scientific).

The amplification program consisted of an initial denaturation at 95 °C for 2 min, followed by 40 cycles of denaturation at 95 °C for 5 s and annealing/extension at 60 °C for 30 s. Relative gene expression levels were calculated using the 2^−ΔΔCt^ method, with β-actin used as the internal reference gene. Primer sequences are listed in Supplementary Table S1.

### 2.8. Effects of FS4722-Fs, FS4722-Dbs, and FS4722-Bl on the MAPK and NF-κB Signaling Pathways

#### 2.8.1. RT–PCR

The effects of FS4722-Fs, FS4722-Dbs, and FS4722-Bl on the MAPK and NF-κB signaling pathways in LPS-stimulated RAW 264.7 macrophages were analyzed by RT–qPCR based on a previously reported method [22] with minor modifications. RAW 264.7 cells (1 × 10^6^ cells/well) were seeded into six-well plates (Corning) and maintained at 37 °C in a humidified atmosphere containing 5% CO_2_ for 24 h. The cells were then treated with the indicated samples for 2 h, followed by stimulation with or without LPS (1 μg/mL) for an additional 24 h.

Total RNA was extracted using TRIzol reagent according to the manufacturer’s instructions. RNA purity and concentration were determined using a NanoPhotometer^®^ N60 spectrophotometer (Implen). Subsequently, 1 μg of total RNA was reverse-transcribed into cDNA using the SensiFAST™ cDNA Synthesis Kit (Nanjing Jiancheng Bioengineering Institute). Quantitative PCR was performed using the SensiFAST™ SYBR No-ROX Kit (Nanjing Jiancheng Bioengineering Institute) with SYBR Green chemistry on a QuantStudio™ 1 Real-Time PCR System (Thermo Fisher Scientific).

The amplification program consisted of an initial denaturation at 95 °C for 2 min, followed by 40 cycles of denaturation at 95 °C for 5 s and annealing/extension at 60 °C for 30 s. Relative gene expression levels were calculated using the 2^−ΔΔCt^ method with β-actin as the internal reference gene. Primer sequences used for amplification are provided in Supplementary Table S2.

#### 2.8.2. Western Blot Analysis

Western blot analysis was performed to evaluate the activation of key pro-inflammatory signaling proteins in LPS-stimulated RAW 264.7 macrophages based on a previously reported method [21] with minor modifications. RAW 264.7 cells (1 × 10^6^ cells/well) were seeded into six-well plates and treated with heat-killed lactic acid bacteria (LAB) strains for 2 h, followed by stimulation with or without LPS (1 μg/mL).

After treatment, the cells were washed twice with ice-cold phosphate-buffered saline (PBS; Nanjing Jiancheng Bioengineering Institute) and subsequently lysed using Pro-prep lysis buffer supplemented with protease and phosphatase inhibitor cocktails. Protein concentrations were determined using a DC™ Protein Assay Kit (Nanjing Jiancheng Bioengineering Institute). Equal amounts of protein (20 μg) were separated by SDS–PAGE and electrotransferred onto PVDF membranes.

The membranes were incubated overnight at 4 °C with primary antibodies against MyD88, phosphorylated ERK1/2 (p-ERK1/2), ERK1/2, phosphorylated JNK (p-JNK), JNK, phosphorylated p38 (p-p38), p38, phosphorylated IκB-α (p-IκB-α), IκB-α, phosphorylated p65 (p-p65), p65, and β-actin. After washing with Tris-buffered saline containing 0.1% Tween-20 (TBST), the membranes were incubated with horseradish peroxidase (HRP)-conjugated secondary antibodies for 1 h at room temperature. Protein bands were visualized using an ECL detection system and recorded on X-ray film. Band intensities were quantified using ImageJ software (National Institutes of Health, Bethesda, MD, USA), and the expression levels of target proteins were normalized to β-actin.

### 2.9. Optimization of the FS4722 Freeze-Drying Process

#### 2.9.1. Single-Factor Experiments

A systematic comparison of different types and concentrations of cryoprotectants was conducted to evaluate their effects on the survival of *L. plantarum* FS4722. The survival rate of the strain after freeze-drying and rehydration was used as the evaluation index. Briefly, 0.1 g of freeze-dried bacterial powder was accurately weighed and resuspended in 0.9 mL of sterile distilled water. The rehydrated suspension was serially diluted in 10-fold increments, and 100 μL of the appropriate dilution was evenly spread onto the surface of MRS agar plates using a sterile spreader. The inoculated plates were incubated at 37 °C for 48 h. Subsequently, the number of colonies was counted to determine the viable cell concentration after freeze-drying. The freeze-drying survival rate (%) was calculated using the following equation:

$$\mathrm{Survival}\;\mathrm{rate}\;(\%)\;=\frac{N1\;}{N0}\times \;1$$

N_1_ is the number of cells that lived through freeze-drying (CFU/mL), and N_0_ is the number of cells that were alive before freeze-drying (CFU/mL).

#### 2.9.2. Response Surface Methodology (RSM) Optimization Experiment

Using Design-Expert 10 software (Stat-Ease Inc., Minneapolis, MN, USA), the results of the single-factor experiment led to the use of RSM. Table S3 shows the experimental design matrix. The main things that were looked at were A (powdered skim milk), B (trehalose), and C (sodium carboxymethyl cellulose), and how they worked together to affect the survival rate of freeze-drying *L. plantarum* FS4722. The model produced an F-value of 3.86 with marginal significance (*p* = 0.0497), indicating that while it explains a significant amount of variation in survival rates, unmeasured interactive effects and inherent biological variability in bacterial stress responses may limit predictive accuracy. This marginal significance suggests that confirmatory experiments are essential for thorough interpretation and validation.

### 2.10. Structuralist Analysis of L. plantarum FS4722 Freeze-Dried Powder

Scanning electron microscopy (SEM) examination of *L. plantarum* FS4722 freeze-dried powder was conducted following a previously established methodology. The surface microstructure of the powder was examined using a field-emission scanning electron microscope (S4800; Hitachi, Tokyo, Japan) at an accelerating voltage of 10.0 kV.

### 2.11. Storage Stability Analysis of L. plantarum FS4722 Freeze-Dried Powder

Freeze-dried bacterial powders with or without the optimal composite cryoprotectant were packaged in polyethylene sterile sampling bags (WHIRL-PAK^®^, Nasco, Fort Atkinson, WI, USA) and stored at 4 °C in a constant-temperature incubator (Model ICH110; Memmert, Schwabach, Germany). Samples were taken every seven days. In accordance with accepted procedures for initial screening of probiotic formulations, storage stability was assessed over a 28-day period as an expedited stability assessment [23,24]. Although commercial products usually aim for shelf-lives of 12 to 24 months, this period allows for quick optimization of cryoprotectant formulations and the detection of stability trends; industrial scale-up necessitates longer-term validation under a variety of conditions. One gram of powder was accurately weighed and resuspended in 9 mL of sterile distilled water, followed by serial 10-fold dilutions. An appropriate dilution was plated onto MRS agar plates and incubated at 37 °C for 48 h for colony enumeration. Storage stability was evaluated based on changes in the logarithmic viable cell counts at different storage times after freeze-drying.

### 2.12. Statistical Analysis

All experiments were performed independently in triplicate, and the results are presented as the mean ± standard deviation (SD). Experimental design and response surface methodology (RSM) analysis were conducted using Design-Expert software (version 10.0; Stat-Ease Inc., Minneapolis, MN, USA). One-way analysis of variance (ANOVA) was performed using SPSS software (version 20.0; IBM Corp., Armonk, NY, USA), followed by Duncan’s multiple range test for post hoc comparisons. Differences were considered statistically significant at *p* < 0.05 and highly significant at *p* < 0.01. Statistical significance is indicated as *p* < 0.05 (*), *p* < 0.01 (**), and *p* < 0.001 (***).

## 3. Results and Discussion

### 3.1. Inhibition of LPS-Induced NO Production Without Cytotoxicity

**Figure 1:**
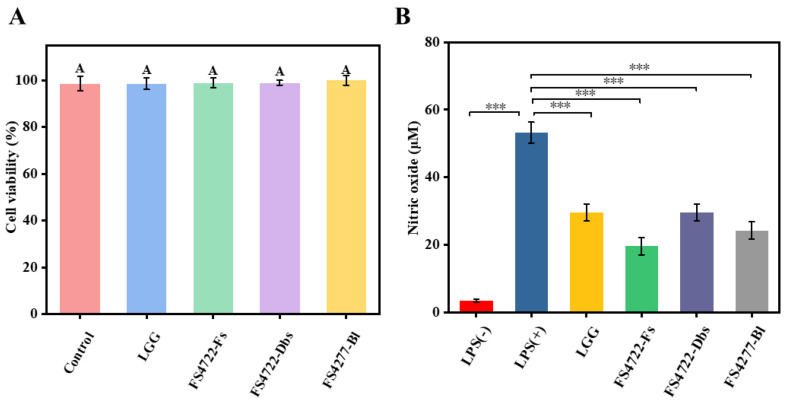
(**A**) Cytotoxicity assessment of FS4722-Fs, FS4722-Dbs, and FS4722-Bl in RAW 264.7 macrophages. (**B**) Inhibitory effects of FS4722-Fs, FS4722-Dbs, and FS4722-Bl on LPS-induced NO production in RAW 264.7 cells. *p* < 0.001 (***). A indicates that there was no significant difference in cell activity among different groups (*p* > 0.05).

To ascertain if the anti-inflammatory effects were due to cytotoxicity, we initially assessed the impact of FS4722-Fs, FS4722-Dbs, andFS4722-Bl on whether RAW 264.7 cells can survive. I verified cell viability and ruled out cytotoxicity as a confounding factor using the MTT assay. Over 95% of cells remained viable in the tested concentration range for all three treatments: FS4722-Fs (97.3 ± 2.1%), FS4722-Dbs (96.8 ± 1.8%), and FS4722-Bl (95.4 ± 2.4%) (Figure 1A). Treated cells did not differ significantly from untreated controls (*p* > 0.05). These results confirm that these dosages are non-cytotoxic and suitable for subsequent anti-inflammatory mechanism studies.

The anti-inflammatory effect of *L. plantarum* FS4722 was evaluated by measuring NO production in LPS-stimulated RAW 264.7 cells (Figure 1B). Compared to the group that did not receive LPS treatment, the LPS-treated group showed a significant increase in NO production and release (*p* > 0.05). LPS stimulates the TLR4/NF-κB signaling pathway, which makes this response happen by increasing the production of inducible nitric oxide synthase (iNOS) [25]. Nonetheless, pretreatment with FS4722-Fs, FS4722-Dbs, or FS4722-Bl markedly inhibited LPS-induced NO generation (*p* > 0.05). FS4722-Fs had the strongest inhibitory effect, surpassing both FS4722-Dbs and FS4722-Bl. A recent study conducted by Cruz-Valencia et al. [26] identified soluble bioactive metabolites from probiotic strains, such as short-chain fatty acids, exopolysaccharides, and peptides, that can have strong anti-inflammatory effects by either directly inhibiting the activity of the iNOS enzyme or blocking NF-κB nuclear translocation. Our results align with this mechanism and indicate that the metabolites concentrated in FS4722-Fs may enhance its efficacy. Moreover, in comparison to the commercial probiotic reference strain LGG, both FS4722-Fs and FS4722-Bl exhibited markedly reduced NO production, indicating their enhanced capacity to modulate inflammatory responses.

### 3.2. Effect of FS4722 on the mRNA Expression of Inflammation-Related Genes

**Figure 2:**
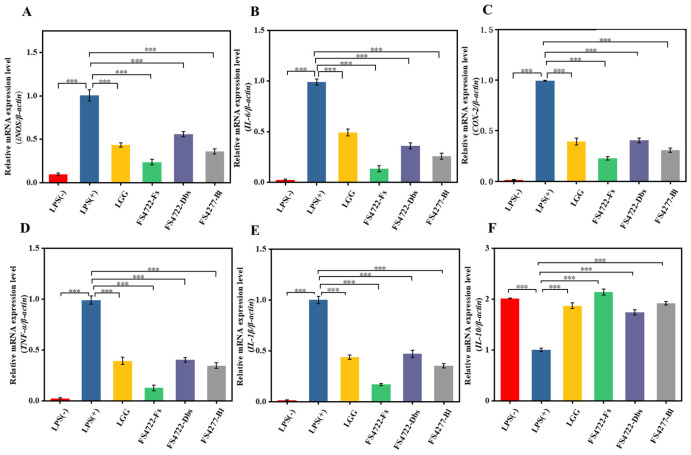
FS4722 changes stop the mRNA expression of important inflammatory mediators in RAW 264.7 macrophages that have been activated by LPS: (**A**) iNOS, (**B**) IL-6, (**C**) COX-2, (**D**) TNF-α, (**E**) IL-1β, and (**F**) IL-10 following treatment with FS4722-Fs, FS4722-Dbs, or FS4722-Bl and LPS stimulation. *p* < 0.001 (***).

Changes in the mRNA expression levels of key inflammatory mediators were analyzed by RT–qPCR to evaluate the transcriptional regulatory effects of FS4722 on the inflammatory response. The results demonstrated that LPS stimulation markedly elevated the transcriptional levels of all target genes (Figure 2A–F). Nonetheless, pretreatment with FS4722-Fs, FS4722-Dbs, or FS4722-Bl significantly reduced the mRNA expression of pro-inflammatory mediators (iNOS, COX-2) and cytokines (TNF-α, IL-6), while markedly increasing the transcript level of the anti-inflammatory cytokine IL-10.

FS4722-Fs exhibited the greatest inhibitory effect on pro-inflammatory genes and the highest induction of IL-10, with suppression rates of 46.67% for iNOS, 52.34% for COX-2, 48.92% for TNF-α, 51.27% for IL-6, and 44.15% for IL-1β, and a 3.2-fold increase in IL-10 compared to the LPS control [26]. The anti-inflammatory effects are attributed to bioactive compounds (e.g., short-chain fatty acids, exopolysaccharides, peptides) that interrupt TLR4-mediated signaling and prevent NF-κB activation, thereby reducing pro-inflammatory gene expression and increasing IL-10 production [26,27]. In summary, while our data clearly establish that FS4722-Fs contains potent immunomodulatory activity, the precise molecular entities and their cognate receptors remain to be identified.

### 3.3. Impact of FS4722 on the Protein Expression of Inflammatory Cytokines in LPS-Induced RAW 264.7 Cells

**Figure 3:**
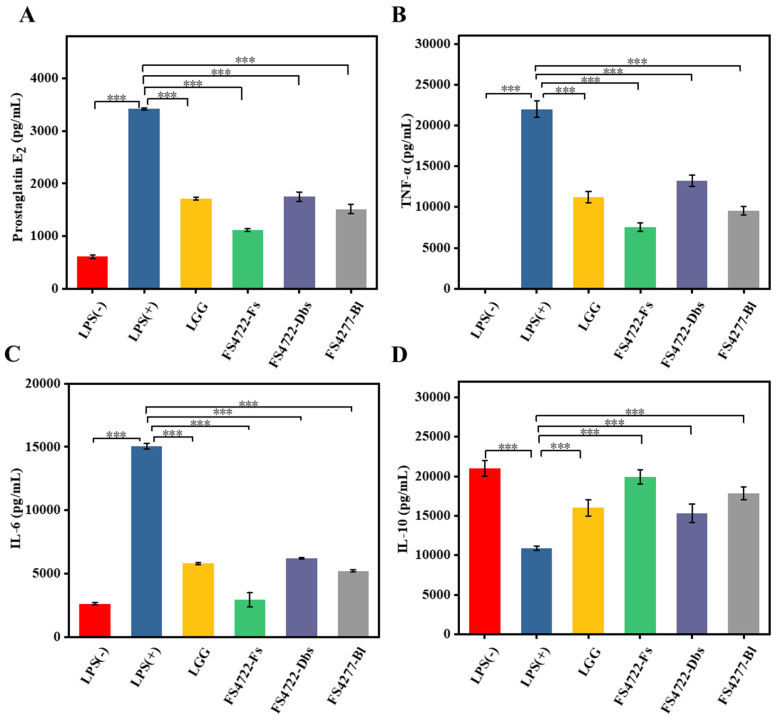
Production of inflammatory mediators and cytokines in LPS-activated RAW 264.7 cells treated with FS4722-Fs, FS4722-Dbs, and FS4722-Bl. The ELISA results for (**A**) PGE2, (**B**) TNF-α, (**C**) IL-6, and (**D**) IL-10 are displayed. *p* < 0.001 (***).

The effects of FS4722-Fs, FS4722-Dbs, and FS4722-Bl on the release of inflammatory cytokines in LPS-stimulated RAW 264.7 cells were evaluated using ELISA. LPS stimulation significantly increased the release of PGE2, TNF-α, and IL-6 (*p* < 0.05), while simultaneously suppressing the production of the anti-inflammatory cytokine IL-10, as seen in Figure 3A–D. Pretreatment with *L. plantarum* FS4722 substantially reduced the protein expression of pro-inflammatory factors (PGE2, TNF-α, and IL-6) and restored IL-10 levels. Lee et al. [21] conducted a study on *L. plantarum* WB3801 and WB3802, revealing analogous results that indicate these strains regulate the synthesis and secretion of inflammatory cytokines at both transcriptional and post-transcriptional levels. Significantly, in comparison to the commercial probiotic reference strain LGG, all *L. plantarum* FS4722 treatment groups demonstrated comparable or superior efficacy in suppressing the expression of pro-inflammatory cytokines.

### 3.4. FS4722′s Impacts on the MAPK and MyD88/NF-κB Signaling Pathways in RAW 264.7 Cells That Were Caused by LPS

To elucidate the anti-inflammatory mechanisms of *L. plantarum* FS4722, RT-qPCR was employed to comprehensively assess the mRNA expression of pivotal genes within the MAPK and MyD88/NF-κB signaling pathways in LPS-stimulated RAW 264.7 macrophages. (Supplementary Figure S1A–F) LPS stimulation made the mRNA levels of ERK1/2, JNK, and p38 in the MAPK pathway much higher than in the control group. Treatment with FS4722-Fs, FS4722-Dbs, or FS4722-Bl significantly diminished the expression of these genes. FS4722-Fs had the most pronounced inhibitory effect among the examined groups, with suppression rates of 46.67%, 49.39%, and 34.04% for ERK1/2, JNK, and p38 mRNA, respectively, in contrast to the commercial probiotic LGG (*p* < 0.05).

In the MyD88/NF-κB signaling system, LPS stimulation similarly resulted in a substantial elevation in the mRNA expression of MyD88, IκB-α, and p65 (Figure S1D–F), hence validating the successful activation of the NF-κB pathway. Conversely, treatment with the FS4722 markedly inhibited the transcriptional activation of these genes, with FS4722-Fs demonstrating the most substantial suppressive effect. This suppression may be due to bioactive compounds in FS4722, like short-chain fatty acids and exopolysaccharides, which may stop the TLR4/MyD88 complex from forming, which would stop IκB-α from breaking down and p65 from moving into the nucleus, which would ultimately stop NF-κB signaling [27]. FS4722-Fs achieved suppression rates of 46.67% (ERK1/2), 49.39% (JNK), 34.04% (p38), 34.83% (MyD88), 27.20% (IκB-α), and 24.04% (p65), exceeding LGG inhibition for all targets (*p* < 0.05).

**Figure 4:**
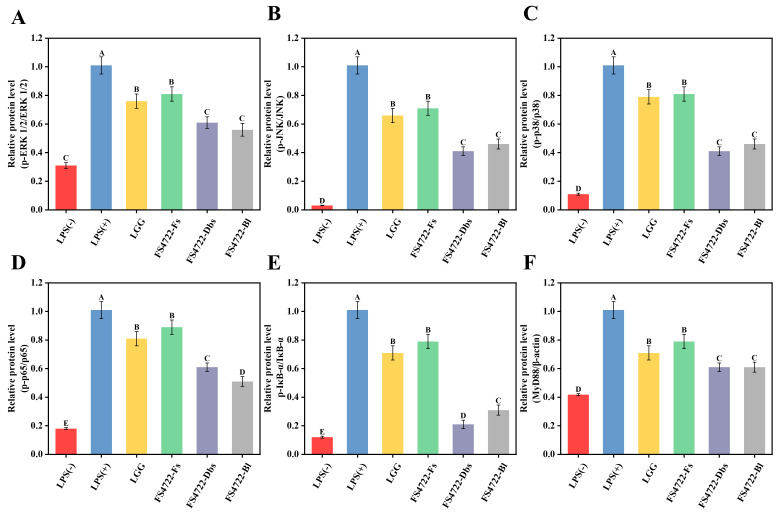
The effect of FS4722 on the MAPK and MyD88/NF-κB signaling pathways in LPS-activated RAW 264.7 cells. The amounts of (**A**) p-ERK 1/2 and ERK 1/2, (**B**) p-JNK and JNK, (**C**) p-p38 and p38, (**D**) p-p65 protein and p65, (**E**) p-IκB-α and IκB-α, (**F**) MyD88 and GAPDH proteins are compared to each other. The statistics are displayed as the mean plus or minus the standard deviation from three distinct investigations. Different letters indicate that the disparities among groups are statistically significant (*p* < 0.05).

To assess the stability of these transcriptional modifications at the protein level and clarify FS4722’s regulatory effect on inflammation-related signaling pathways, Western blot analysis was performed to measure the expression of MAPK- and MyD88/NF-κB–associated proteins in LPS-stimulated RAW 264.7 cells. Compared to the untreated group, LPS raised the levels of the proteins p-ERK1/2, p-JNK, and p-p38 significantly. Figure 4A–C demonstrates that the application of heat-killed bacterial samples significantly diminished the phosphorylation levels of ERK1/2, JNK, and p38 across all groups. FS4722-Fs effectively reversed the LPS-induced phosphorylation of MyD88, IκB-α, and p65 within the MyD88/NF-κB pathway (Figure 4D–F). In line with the MAPK findings, FS4722-Fs demonstrated the most significant inhibitory effects on protein phosphorylation.

These data jointly demonstrate that FS4722-Fs synergistically inhibit the aberrant activation of the MAPK and MyD88/NF-κB signaling pathways at both the transcriptional and protein phosphorylation levels, thereby effectively mitigating LPS-induced inflammatory responses. These findings furnish extensive molecular evidence corroborating the prospective anti-inflammatory mechanism of FS4722-Fs.

### 3.5. Preliminary Screening of Single Cryoprotectants for L. plantarum FS4722

**Figure 5:**
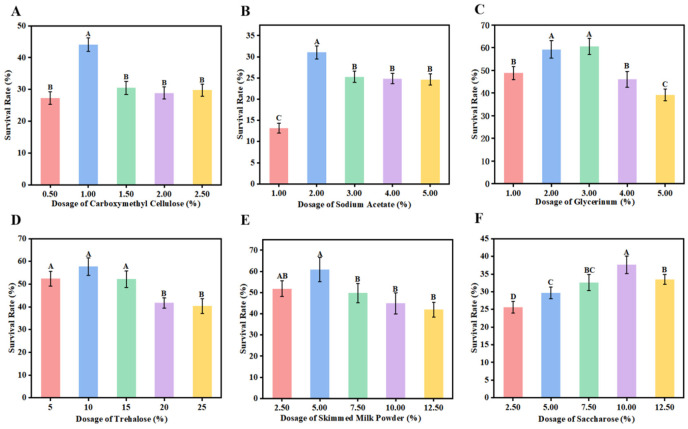
The impact of freeze-drying protectants on the viability of Lactobacillus plantarum. (**A**) Sodium carboxymethyl cellulose; (**B**) Sodium acetate; (**C**) Glycerol; (**D**) Trehalose; (**E**) Skim milk powder; (**F**) Sucrose. The data are shown as mean ± SD (n = 3). The bars have different capital letters above them, which means that there are big differences (*p* < 0.05).

This study thoroughly investigated the protective powers of various agents on the survival of *L. plantarum* FS4722 after lyophilization to see how different cryoprotectants affect how well the strain can handle freeze-drying. The findings indicated that various cryoprotectants markedly enhanced survivability during freeze-drying via unique mechanisms. Figure 5A shows that CMC-Na had protective effects in the 0.50–2.50% range, with the strongest effect at 1.00%, which led to a survival rate of 44.07%, which is about 2.3 times greater than that of the blank group. The polymeric network created by CMC-Na is mostly responsible for this protection. It stops ice crystals from forming, reduces mechanical damage, and keeps the cell membrane stable while it is drying out.

Sodium acetate also offered protection between 1.00% and 5.00% (Figure 5B). The highest survival rate (31.03%) was attained with a 2.00% addition, which was significantly different from the control group (*p* < 0.05). The mechanism might be linked to the pH buffering ability of sodium acetate, which aids in preserving intra- and extracellular pH stability during lyophilization, consequently mitigating protein denaturation damage [28]. Glycerol demonstrated the most effective protective influence among all cryoprotectants (Figure 5C). At a concentration of 3.00%, the survival rate attained 60.47%, markedly above that of other groups (*p* < 0.01). Glycerol is a penetrating cryoprotectant that enters cells, lowers the freezing point, stops huge ice crystals from forming, and replaces water molecules by hydrogen bonding. This keeps the structure of proteins [29]. Adding 10.00% trehalose led to a survival rate of 57.71% (Figure 5D). Its protective function derives from its robust glass-forming properties, which create an amorphous glassy matrix on the cell surface that stabilizes membrane lipids via hydrogen bonding and promotes the restoration of membrane fluidity upon rehydration [30]. At a concentration of 5.00%, skim milk powder also provided significant protection, with a survival percentage of 60.85% (Figure 5E). This impact is due to its composite composition: milk proteins stick to the surface of bacteria through hydrophobic interactions to create a physical barrier, while lactose helps keep the structure of cells intact through glass transition [31].

At a concentration of 10.00% sucrose, the survival rate was 37.64% (Figure 5F). Even though it was much higher than the blank control *(p* < 0.05), its protective effect was weaker than that of trehalose. This is likely because sucrose has a lower glass transition temperature. The process also involves the formation of a glassy matrix that restricts molecular mobility and provides protection [32].

Our findings indicate that glycerol, trehalose, CMC-Na, and skim milk powder are effective cryoprotectants that can considerably improve the survival of *L. plantarum* FS4722 during freeze-drying. Their protective mechanisms involve several processes, including glassy matrix formation, osmotic regulation, and membrane stabilization, providing a theoretical foundation for the development of highly effective lyoprotectant formulations. However, since *L. plantarum* FS4722 products are intended for consumption, the use of glycerol may raise safety concerns, highlighting the importance of selecting food-grade excipients. Accordingly, the combination of skim milk powder, trehalose, and CMC-Na emerges as the most suitable cryoprotectant system for this strain, balancing both efficacy and safety.

### 3.6. Optimization of the Cryoprotectant Formulation for L. plantarum FS4722 Using RSM

Using the single-factor experiments, RSM was applied to examine how different amounts of trehalose, CMC-Na, and skim milk powder affected the survival rate of *L. plantarum* FS4722 after freeze-drying, as presented in Table S4. The model produced an F-value of 3.86 and a *p*-value of 0.0497 (*p* < 0.05), as shown in Table S5. This indicates that the model is statistically significant and appropriately reflects how the factors influence the survival rate (response value). The model showed marginal significance (F = 3.86, *p* = 0.0497), with skim milk powder exerting the strongest linear effect on survival rate, followed by trehalose and CMC-Na, suggesting that its predictive accuracy may be limited.

**Figure 6:**
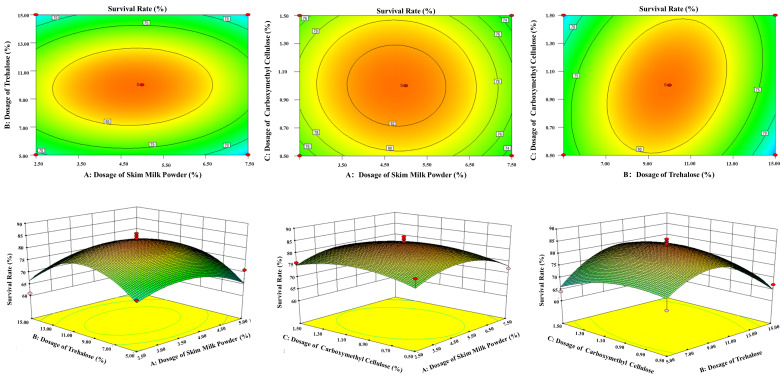
The three-dimensional response surfaces show that different freeze-drying protectants changed how well *L. plantarum* FS4722 survived. **A** Powdered skim milk; **B** Trehalose; **C** Sodium carboxymethyl cellulose.

Analysis of the linear and quadratic coefficients for each component revealed that the concentration of skim milk powder had the greatest effect on the response value, followed by trehalose and then CMC-Na. RSM was used to look at how factors interacted with each other, and Figure 6 illustrates the impact of each factor on bacterial survival, presented in three-dimensional response surfaces and two-dimensional contour plots. The Design-Expert program estimated that the optimal cryoprotectant formulation consists of 5.00% skim milk powder, 10.00% trehalose, and 1.00% CMC-Na. Under these optimal conditions, *L. plantarum* FS4722 achieved a survival rate of 82.33% after freeze-drying. Statistical analysis confirmed no significant difference between this value and the peak survival rates achieved with other tested combinations, confirming the formulation’s robustness.

### 3.7. Microstructural Analysis of L. plantarum FS4722 Freeze-Dried Powder

**Figure 7:**
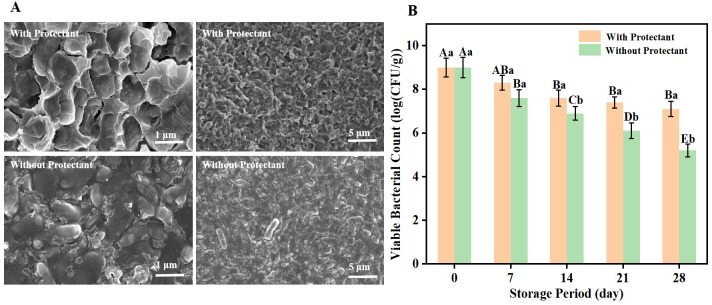
(**A**) SEM pictures of freeze-dried powder of *L. plantarum* FS4722 with and without protectants. (**B**) The effect of composite protective agents on the viability of *L. plantarum* FS4722 in the freeze-dried state. The data are presented as mean ± SD (n = 3). Different uppercase letters above the vertical lines show that there are big variations (*p* < 0.05) between the different storage timeframes. The use of different lowercase letters shows that there are big differences (*p* < 0.05) between the groups that had protectants and those that didn’t at the same time.

Scanning electron microscopy (SEM) was employed to investigate the microstructure of freeze-dried *L. plantarum* FS4722 powders, both with and without cryoprotectants. SEM examination revealed intact cell morphology with defined membrane structures in cryoprotectant-treated samples (Figure 7A). Samples without cryoprotectants showed membrane disruption, morphological distortion, and apparent intracellular content leakage, such as trehalose or sucrose, forming a glassy or amorphous matrix during freezing and drying. This keeps the phospholipid bilayer of the cell membrane stable and lowers the damage caused by ice crystals and osmotic stress [30,32].

On the other hand, samples made without cryoprotectants (Figure 7A) showed a lot of structural damage, such as loss of membrane integrity, changes in shape, and what looked like leaking of intracellular contents. This is mainly because intracellular free water escapes quickly, and ice crystals grow quickly during fast freezing and vacuum drying. This causes membrane lipid denaturation, protein inactivation, and cell wall collapse, which leads to permanent structural damage [33]. These results show that *L. plantarum* FS4722 needs a lot of cryoprotectants to keep its structure and biological activity after lyophilization. For the production of highly viable probiotic formulations, it is very important to improve the cryoprotectant formulation and freeze-drying procedure.

### 3.8. Effect of Freeze-Drying Protectants on the Storage Stability of L. plantarum FS4722 Powder

Variations in viable cell counts during storage at 4 °C were systematically assessed to evaluate the effect of the composite cryoprotectant formulation on the storage stability of *L. plantarum* FS4722. While being kept at 4 °C, the number of cells that could live was tracked (Figure 7B). At 7 days, the vitality of the cryoprotectant-treated group declined initially (*p* < 0.05), but stabilized by day 14, showing no further significant alterations (*p* > 0.05). The group that didn’t get treatment showed a continuous reduction in viability that happened during lyophilization [23]. The protectants did a good job of reducing the damage, but the cells had to go through a short recovery phase, which caused temporary changes in their viability.

After 14 days of storage, the viable cell count in the cryoprotectant group reached a stable level, with no further significant changes observed (*p* > 0.05). This was due to the formation of a stable glassy matrix, aided by components like trehalose and skim milk powder, which effectively suppressed molecular mobility and residual biochemical activity [24]. This put the cells’ metabolism almost to sleep, ensuring they would remain stable for a long time. The group without protectant, on the other hand, showed a steady reduction in viability over the storage time. This loss was due to permanent and cumulative damage, such as membrane lipid peroxidation, protein denaturation, and DNA damage, produced by long-term exposure to environmental stressors such as oxygen, humidity, and changes in temperature [34]. The cryoprotectant group had a lot more viable cells than the control group by day 14. During this time period, control cells had died a lot since they had been exposed to stress for a long time. On the other hand, cryoprotected cells, after an initial phase of adaptation, showed clear stability benefits, staying alive at a rather high plateau level. As a result, a statistically significant difference was observed between the two groups.

These findings indicate that the formulated composite cryoprotectant effectively protects cells from external stresses by creating a protective glassy matrix, thereby markedly improving the survival and stability of *L. plantarum* FS4722 during prolonged storage. This study provides essential technological and theoretical support for its industrial implementation and the development of high-viability freeze-dried bacterial powder formulations.

## 4. Conclusions

This study elucidates the dual functionality of *L. plantarum* FS4722, demonstrating both its anti-inflammatory effects and the optimization of its lyophilization stability for translational applications. Our findings demonstrate that the fermentation supernatant of FS4722 exerts a potent immunomodulatory effect in LPS-stimulated RAW 264.7 macrophages, primarily by attenuating the MAPK signaling pathway and inhibiting MyD88-dependent NF-κB activation, thereby suppressing the transcription of pro-inflammatory cytokines and mediators. Concurrently, we developed a composite cryoprotectant formulation—comprising trehalose, sodium carboxymethyl cellulose, and skim milk—that significantly enhances post-lyophilization viability, preserves cellular morphology, and ensures stability during storage at 4 °C.

Despite these advances, we acknowledge several limitations that contextualize our findings and open avenues for future research. First, the mechanistic insights reported here are derived exclusively from an in vitro murine macrophage model, which, while informative, cannot fully recapitulate the complex cellular crosstalk and microenvironment of the intestinal immune system in vivo. Second, while we identified the involvement of the MAPK/NF-κB axes, the specific molecular moieties within the FS4722 fermentation supernatant responsible for this bioactivity remain unidentified, precluding a complete understanding of the ligand–receptor interactions at play. Third, although the optimized lyoprotectant ensures cellular integrity during storage, the functional efficacy of the reconstituted probiotics—particularly their capacity to colonize and exert anti-inflammatory effects in a gastrointestinal environment—has yet to be validated.

In summary, this work provides a critical theoretical and technical foundation for the industrial development of *L. plantarum* FS4722-based anti-inflammatory products. However, translating these findings into practical applications will necessitate future investigations focused on: (i) identifying the active bioactive compounds through metabolomic profiling, (ii) validating the anti-inflammatory efficacy and safety in appropriate animal models of colitis, and (iii) assessing the long-term functional stability of the lyophilized formulation under simulated gastrointestinal conditions.

## Data Availability

The original contributions presented in this study are included in the article/Supplementary Materials. Further inquiries can be directed to the corresponding authors.

## Supplementary Materials

The following supporting information can be downloaded at: https://www.mdpi.com/article/10.3390/foods15061096/s1, Figure S1. Effects of the MAPK and MyD88/NF-κB pathway in LPS-stimulated RAW 264.7 cells treated with FS4722-Fs, FS4722-Dbs, and FS4722-Bl. Relative mRNA levels of (A) ERK 1/2, (B) JNK, (C) p38, (D) MyD88, (E) IκB, and (F) p65. Data are presented as mean ± SD (n = 3). * *p* < 0.05, ** *p* < 0.01, *** *p* < 0.001 versus LPS (+) group by one-way ANOVA with Dunnett’s post hoc test. Table S1: Primers sequences for real-time reverse transcription-polymerase chain reaction (RT-PCR). Table S2: Primers sequences for real-time reverse transcription-polymerase chain reaction (RT-PCR). Table S3. Response Surface Test Factor Levels. Table S4. RSE Optimization for the Survival of *L. plantarum* FS4722. Table S5. ANOVA for the survival rate of *L. plantarum* FS4722.
