# Supplementary material for: Anti-Inflammatory Effects of Lactiplantibacillus plantarum Strain FS4722 Through MAPK and NF-κB Signaling Pathways and Its Lyophilization Optimization
Source: Foods. 2026 Mar 20;15(6):1096. doi: 10.3390/foods15061096 (PMC13025716; doi:10.3390/foods15061096)
Supplement: Supplementary file 1 [file foods-15-01096-s001.zip › foods-4152710-supplementary.pdf]

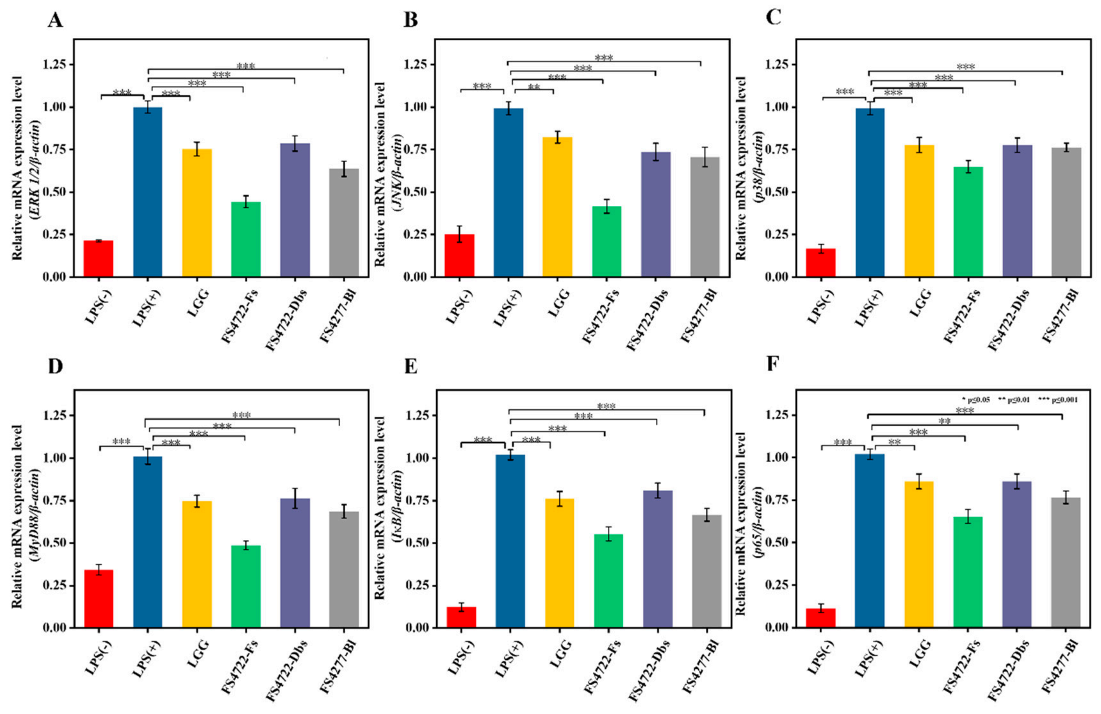

Figure S1: Effects of the MAPK and MyD88/NF-κB pathway in LPS-stimulated RAW 264.7 cells treated with FS4722-Fs, FS4722-Dbs, and FS4722-Bl. Relative mRNA levels of (A) ERK 1/2, (B) JNK, (C) p38, (D) MyD88, (E) IκB, and (F) p65. Data are presented as mean ± SD (n=3). *$p < 0.05$, **$p < 0.01$, ***$p < 0.001$ versus LPS (+) group by one-way ANOVA with Dunnett's post hoc test.

Table S1

Primers sequences for real-time reverse transcription-polymerase chain reaction (RT-PCR).

Primera		Primer sequence (5'–3')
iNOS	Forward	CCCTTCCGAAGTTTCTGGCAGCAGC
	Reverse	GGCTGTCAGAGCCTCGTGGCTTTGG
COX-2	Forward	CACTACATCCTGACCCACTT
	Reverse	ATGCTCCTGCTTGAGTATGT
IL-1β	Forward	CAGGATGAGGACATGAGCACC
	Reverse	CTCTGCAGACTCAAACTCCAC
IL-6	Forward	GTACTCCAGAAGACCAGAGG
	Reverse	TGCTGGTGACAACCACGGCC
TNF-α	Forward	TTGACCTCAGCGCTGAGTTG
	Reverse	CCTGTAGCCCACGTCGTAGC
IL-10	Forward	GACTTTAAGGGTTACCTGGGTTG

	Reverse	TCACATGCGCCTTGATGTCT
β-actin	Forward	GTGGGCCGCCCTAGGCACCAG
	Reverse	GGAGGAAGAGGATGCGGCAGT

Table S2

Primers sequences for real-time reverse transcription-polymerase chain reaction (RT-PCR).

Primera		Primer sequence (5'–3')
ERK 1/2	Forward	CCAGGATTACCTGCCTCAAG
	Reverse	TCTGTAGCCACCACCAATCC
JNK	Forward	GAG AAG GAC TCC CAG CAT GA
	Reverse	GTC AGG GTA GGC AAG GAG TC
P38	Forward	TTC CGA TGG AAC TGT GAC CC
	Reverse	GTT GCT GGC AAT GAG CGA TA
MyD88	Forward	GGA ACT GCA GAC CTG GAA GG
	Reverse	GCT GGT GGA GTT CAA GTC GG
IκB-α	Forward	AGG CCA GGA GAC CAG GAA T
	Reverse	TCC TTG AGC TCA GGA ATC CG
P 65	Forward	AGC ACC AGC TGG TCA GGT AT
	Reverse	TCT GGC TGG ACT CCA TGA TC
β-actin	Forward	GTGGGCCGCCCTAGGCACCAG
	Reverse	GGAGGAAGAGGATGCGGCAGT

Table S3. Response Surface Test Factor Levels.

Factors	Levels		
	-1	0	1
A: Dosage of Skim Milk Powder (%)	2.50	5.00	7.50
B: Dosage of Trehalose (%)	5.00	10.00	15.00
C: Dosage of Carboxymethyl Cellulose (%)	0.50	1.00	1.50

Table S4. RSE Optimization for the Survival of *L. plantarum* FS4722

Experiment Number	A: Dosage of Skim Milk Powder (%)	B: Dosage of Trehalose (%)	C: Dosage of Carboxymethyl Cellulose (%)	Survival Rate (%)
1	7.50	10.00	1.50	69.12±4.19
2	5.00	5.00	0.50	68.24±5.19
3	2.50	5.00	1.00	70.89±3.19
4	7.50	10.00	0.50	72.78±4.18
5	2.50	10.00	1.50	76.67±6.18
6	5.00	10.00	1.00	79.99±6.18
7	7.50	5.00	1.00	71.32±5.18
8	5.00	10.00	1.00	84.19±6.19
9	5.00	10.00	1.00	82.18±7.18
10	5.00	15.00	1.50	76.56±4.18

11	7.50	15.00	1.00	65.44±5.18
12	5.00	5.00	1.50	64.87±4.18
13	2.50	10.00	0.50	78.13±4.18
14	2.50	15.00	1.00	61.56±5.23
15	5.00	10.00	1.00	85.87±6.17
16	5.00	15.00	0.50	67.45±5.34
17	5.00	10.00	1.00	86.92±6.78

Data are presented as mean ± standard deviation of triplicate experiments.

Table S5. ANOVA for the survival rate of *L. plantarum* FS4722

Source of Variance	Sum of Squares	Degrees of Freedom	Mean Square	F-value	p-value	Significance
Model	797.23	9	88.58	3.68	0.0497	significant
A	8.00	1	8.00	0.33	0.5821	
B	2.00	1	2.00	0.083	0.7814	
C	0.000	1	0.000	0.000	1.0000	
AB	2.25	1	2.25	0.094	0.7686	
AC	0.25	1	0.25	0.010	0.9216	
BC	42.25	1	42.25	1.76	0.2266	
A^2	138.00	1	138.00	5.74	0.0478	
B^2	484.32	1	484.32	20.14	0.0028	
C^2	58.42	1	58.42	2.43	0.1630	
Residual	168.30	7	24.04			
Lack of Fit	137.50	3	45.83	5.95	0.0588	not significant

Pure Error	30.80	4	7.70
Cor Total	965.53	16	
